# The WHO costing and budgeting tool for national action plans on antimicrobial resistance—a practical addition to the WHOle toolkit

**DOI:** 10.1093/jacamr/dlad064

**Published:** 2023-05-25

**Authors:** Rebecca E Glover, Nichola R Naylor

**Affiliations:** Department of Health Services Research and Policy, London School of Hygiene and Tropical Medicine, 15-17 Tavistock Place, London WC1H 9SH, UK; UK Health Security Agency, London NW9 5EQ, UK

## Abstract

**Objectives:**

The development of national action plans (NAPs) for antimicrobial resistance (AMR) has been promoted and supported by the WHO, with recent support in the form of costing and budgeting tools to aid in finance-allocation decisions within governments.

**Methods:**

In this brief report we review this WHO costing and budgeting tool, discuss the strengths and weaknesses, and consider its place alongside other health economics and policy-support tools developed.

**Results:**

We call for future analyses of the costs of AMR NAPs to consider costs beyond that of only implementation, through use of other available, open-access data and tools. These include the Global Antimicrobial Resistance and Use Surveillance System (GLASS) data and One Health tools already within the existing ‘WHO toolbox’.

**Conclusions:**

We suggest that future work on evaluating AMR along the impact pipeline use this toolbox where possible, ensuring empirical work is in turn open access.

## Introduction

In 2015, the WHO called on every member country to develop a One Health national action plan (NAP) on antimicrobial resistance (AMR), which, as of October 2021, is a target that has been met by 148 countries.^1,2^ Though many countries have published NAPs, only 20% were fully funded in 2020–21, with a further 40% of NAPs with some budgeted operational plan, leaving a substantial proportion of NAPs unfunded or underfunded. The WHO is endeavouring to facilitate the implementation of NAPs; to that end, in Autumn 2021 they released a costing and budgeting tool.^3^

The tool purports to allow for users to cost technical activities for the AMR NAPs. This tool is joining a suite of other relevant tools and data available from the WHO.^4^ Here, we summarize this new tool (referred to in this paper as the AMR-NAP tool), discuss its potential strengths and weaknesses, and situate this costing and budgeting tool within the context of other evaluation resources available through WHO-funded or associated tools.

## WHO costing and budgeting tool for NAPs on AMR

According to the WHO, the AMR-NAP tool can be used to ‘generate detailed costs for the technical activities included in AMR NAPs. The tool can be used to cost priority activities that still need to be funded.’^3,5^ The technical specifications of the model are developed in Microsoft Excel (in 2010 or later versions), and the tool guides users through the process of inputting cost and financing components, and inflation and currency assumptions, in order to combine and compare cost and budgeting estimates over a 1–5 year period. The backend has more complex functions developed in Visual Basic for Application (VBA). Currently the tool is in English, but with plans to expand to French and Spanish.^3^

Practically, users need to source and calculate unit costs separately (Figure S1b and c, available as Supplementary data at *JAC-AMR* Online). Of note, the ‘building blocks’ for the cost matrices for each activity include ‘meetings’, ‘consultant’, ‘field visit’, ‘HR’, ‘procurement’ and ‘other’. These categories reflect the narrow, activity-based lens of this costing tool. However, potentially incorporating the costs and funding of policies across other health programmes (such as vaccine programmes) is said to be possible.^3^ Different ministries and/or departments can work on individual worksheets to allow for the continuation of siloed, status-quo working, with easier subsequent consolidation on a combined dashboard through the ‘module consolidator’ Excel component.^3^ Although, practically, this tool is sensitive to small deviations from the manual (for example, labelling objectives before setting up the cost matrices leads to error messages), the authors advise intended users to save versions pre- and post- setting up the cost matrices, as otherwise users may have to fill in from scratch to accommodate any changes made to the NAP inputs at a later date.

## Potential strengths and limitations

The WHO’s new tool reflects a drive for local- and national-level NAP prioritization rather than top-down mandates from supranational health governing bodies or high-income countries. This is a step in the right direction for the decolonizing of global health. At first glance, this tool facilitates flexible, country-level control over prioritization exercises and budgeting efforts. It also promotes a stepwise process of rationalization, and allows policy officers to link AMR prioritization efforts to the microeconomic costs of implementing the chosen priority policies.

At a practical level, this tool can be operated using a downloadable Microsoft Excel model, which promotes transparency and consistency within and across settings. Though MS Excel is a paid software, many national governments have taken out subscriptions to this, although this may not be the case at regional- or local-level government. Therefore, we expect the tool to be situated at the national level, as indeed the main messaging from WHO recommends. However, there is likely to be large variation in terms of unit costs (such as salaries or procurement costs) within a country. This may lead to misalignment in costing and funding estimates across local regions in practice, since the tool asks for point estimation of inputs, without the opportunity to input ranges. Scaling up and decentralized funding concerns across ‘subnations’ are highlighted as potential issues with using the tool.^3^ However, individual worksheets can be consolidated across users, which may help technical officers working in AMR across different departments and sectors share information. This may in turn improve resource allocation and coordination.

Additionally, although the tool’s frontend (the user interface) is fit for purpose for the intended end users (policymakers and advisors), it would be helpful for follow-on tools to have clearer backend documentation and navigation (e.g. described VBA queries and macros, alongside methods), to allow for other developers to repurpose tools where necessary. This will also help to reduce research waste.

A broader critique is that the tool specifies that the end user needs to select the order of policy priorities *before* the costing is done. This is important; practically, it is challenging to make the case for prioritization within a government department’s lump sum budget without having an understanding of the cost and how it links to impact. Moreover, although AMR NAPs may be 5 year pre-set commitments, the stated aim of this tool is to help with the policy prioritization and implementation process; the policy decisions of what to prioritize in a particular budget cycle are negotiations and ‘wish lists’, and often iterative processes, so if the tool were to allow for easier comparison across scenarios (and taking into consideration wider impact) that might make it more useful in the process of AMR ‘winning’ policy and budgetary space within the pre-prioritization dialogue.

Technically, the cost matrices are centred around set specific human and capital resources (like laboratory technicians or equipment), but AMR is complex, and the biggest gains in AMR will likely require transformational change at the structural level. This includes water management or health systems strengthening, which are known to be immensely challenging to cost in such a way.^6^ The risk of using such a framework is that the costs and benefits that are not captured may not be advocated for within government, even if these may provide longer-term benefits; rhetoric and attention can be governed by—and govern—the policies that are taken on board^7,8^ However, focused cost inputs and outputs used in—or produced by—the tool could subsequently feed into economic evaluations of intervention impact, so could provide a wider use in costing impact.^9^

## The rest of the toolbox

**Figure 1.**
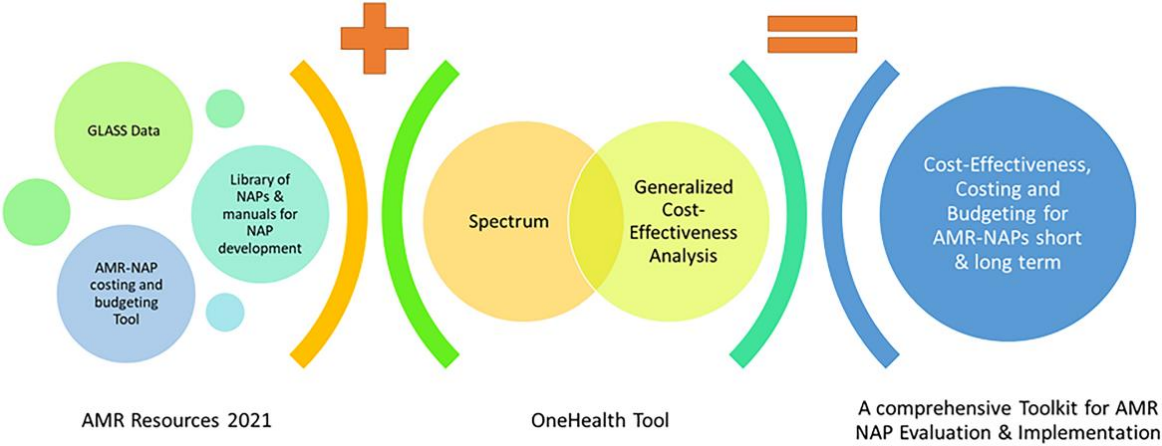
The potential WHOle toolbox for evaluating NAPs for AMR.

This tool exists within a toolbox of other options available to policymakers, including those in Figure 1.^10–13^

The WHO Global Antimicrobial Resistance and Use Surveillance System (GLASS) can provide estimates of AMR and antimicrobial use.^13^ These are needed for flagging priority areas, and in epidemiology and economic models of policy impact. The NAP resources available (including the AMR-NAP tool) provide supporting material for constructing NAPs and calculating the potential costs of policy implementation.^10^ Other WHO tools such as the One Health tool, which has epidemiology (Spectrum) and economic (Generalized Cost-Effectiveness Analysis) modules, provide an overview of the impacts of different scenarios (see Figure S2), although currently only certain diseases, such as TB, report estimates by susceptibility.^11,12^

## Discussion

The AMR-NAP tool provides a useful framework for costing and budgeting. However, its use for post-prioritization and point-estimation means the tool should be considered and used within the wider suite of resources available. AMR is a complex, system-wide problem, and understanding the limitations of such costing tools is important. Whilst it is understandable to focus on what is measurable, policymakers need to be aware that structural, effective interventions may not fall within this remit, and ensure that important considerations do not fall off the policy table simply because they are currently difficult to cost.

## Limitations and recommendations

Fundamentally, when supranational health bodies endeavour to intervene in national government policy development, they should endeavour to create tools that are user friendly, work across multiple interfaces, and truly support countries to adopt the policies and programmes that are right for them. We should consider moving toward open-access and transparent model code and data, which would increase costing and evaluation capacity, and data linkage opportunities, decreasing cost for future tweaks/build-ons to suit need. Although the current tool is compatible with input/output ‘copy and pasting’ with other WHO impact assessment tools, the latter may use different proprietary software, and issues of compatibility with standard in-house budgeting software are already seen with the Microsoft Excel-based tool available.^3^ There may ultimately be a problem of *too many* tools. Although the WHO does provide a tools directory on their website,^4^ it would be useful to highlight when to use which tool across the AMR policy process.

### Conclusions

Overall, this WHO AMR costing and budgeting tool accomplishes its stated aims. This tool should facilitate early-stage AMR NAP efforts for countries that have struggled to make headway in NAP implementation, but is limited in scope for the definition of ‘cost’. By combining this tool with others available, whilst expanding disease-scope of current health and economic impact tools available, national-level capacity in developing, implementing and evaluating AMR NAPs may be maximized.

## Supplementary Material

dlad064_Supplementary_Data

## Transparency declarations

The views expressed are those of the author(s) and are not necessarily those of the National Institute for Health Research, the UK Health Security Agency or the Department of Health and Social Care. Part of N.R.N.’s time has previously been funded by the WHO (in the past 2 years) through grants awarded to the London School of Hygiene & Tropical Medicine on the economic burden of antimicrobial resistance. R.E.G. has previously received funding from WHO SEARO in the form of travel expenses. The authors declare no other conflicts of interest.

## Supplementary data

Figures S1 and S2 are available as Supplementary data at *JAC-AMR* Online.
