# Supplementary material for: The WHO costing and budgeting tool for national action plans on antimicrobial resistance—a practical addition to the WHOle toolkit
Source: JAC Antimicrob Resist. 2023 May 25;5(3):dlad064. doi: 10.1093/jacamr/dlad064 (PMC10210613; doi:10.1093/jacamr/dlad064)
Supplement: dlad064_Supplementary_Data [file dlad064_supplementary_data.docx]

# A Perspective on the WHO Costing and Budgeting Tool for National Action Plans on Antimicrobial Resistance – A practical addition to the WHOle toolkit: Supplementary Material

#### Figure 1. A Test Run of the AMR-NAP Costing Tool

*All values are dummy values within this figure and do not reflect real values*

1. **“National Action Plan Entry” in which users specify the NAP policy and related activities that are to be costed**

**
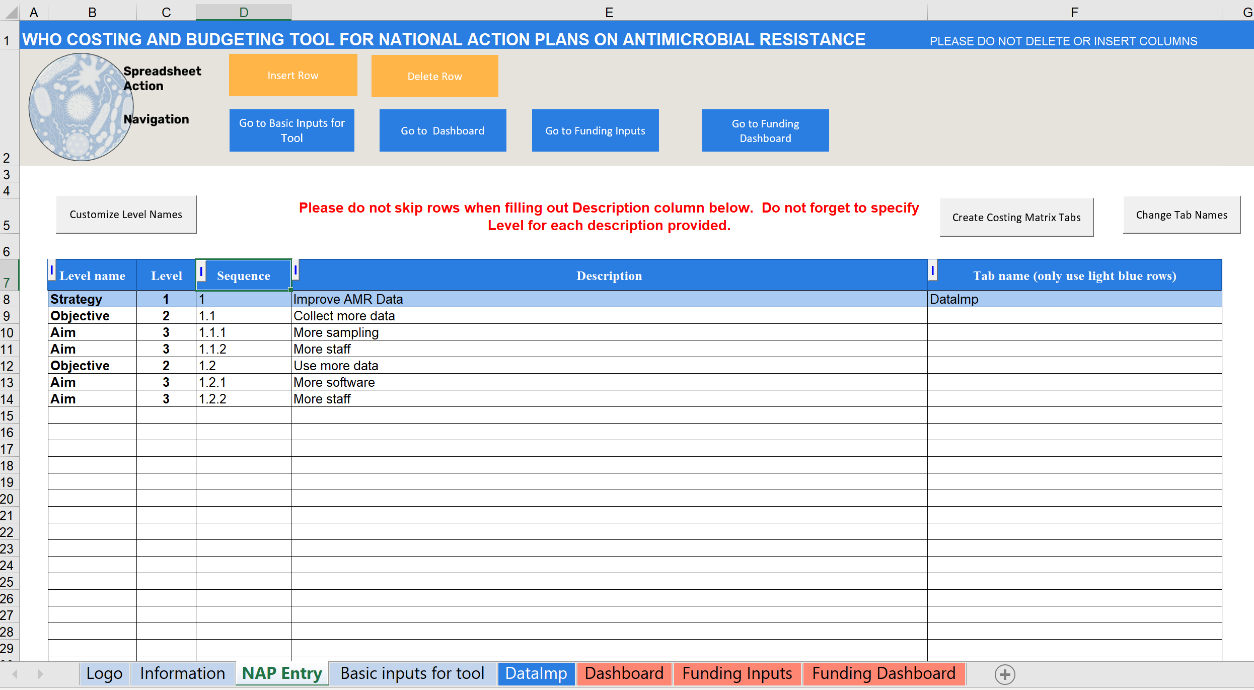
**

1. **“Basic Inputs for the tool” in which users specify the resource use and funding associated with an activity**


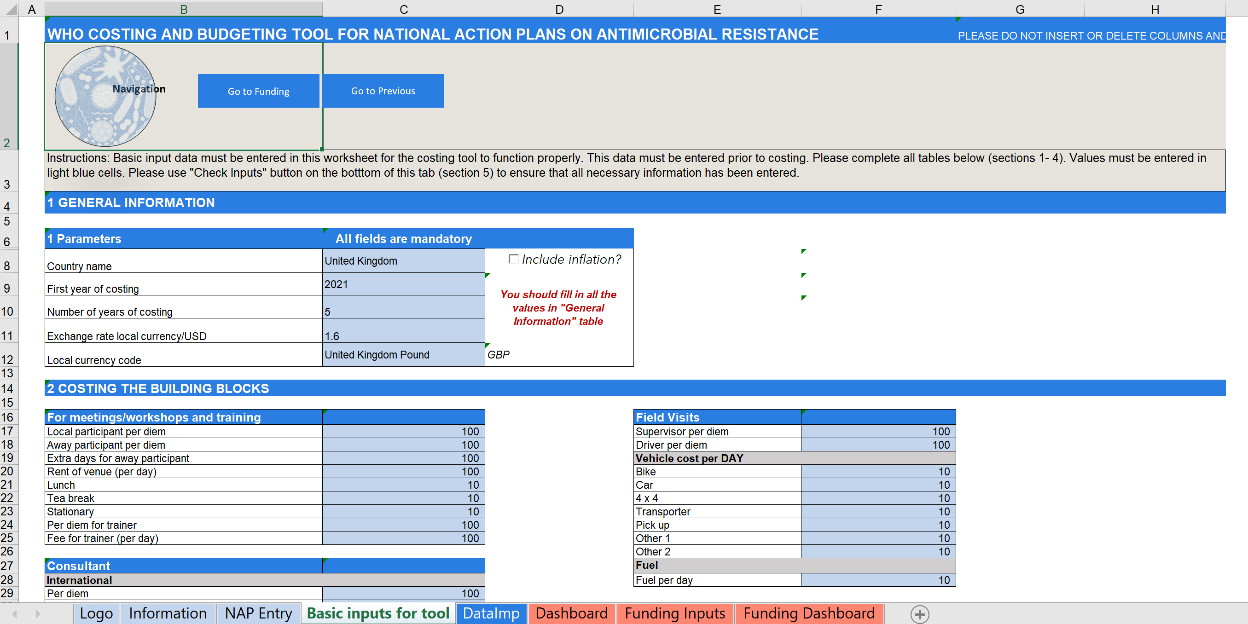


1. **The Cost matrix sheet & building block entry for “HR” in which users specify unit costs**

**
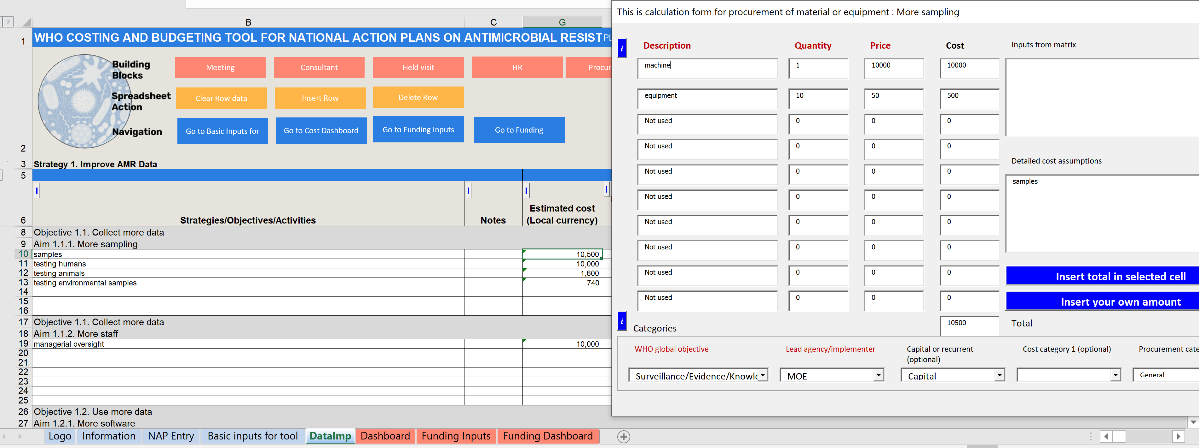
**

1. **Funding Inputs (optional) in which users can specify allocated funding for aims, objectives or strategies**


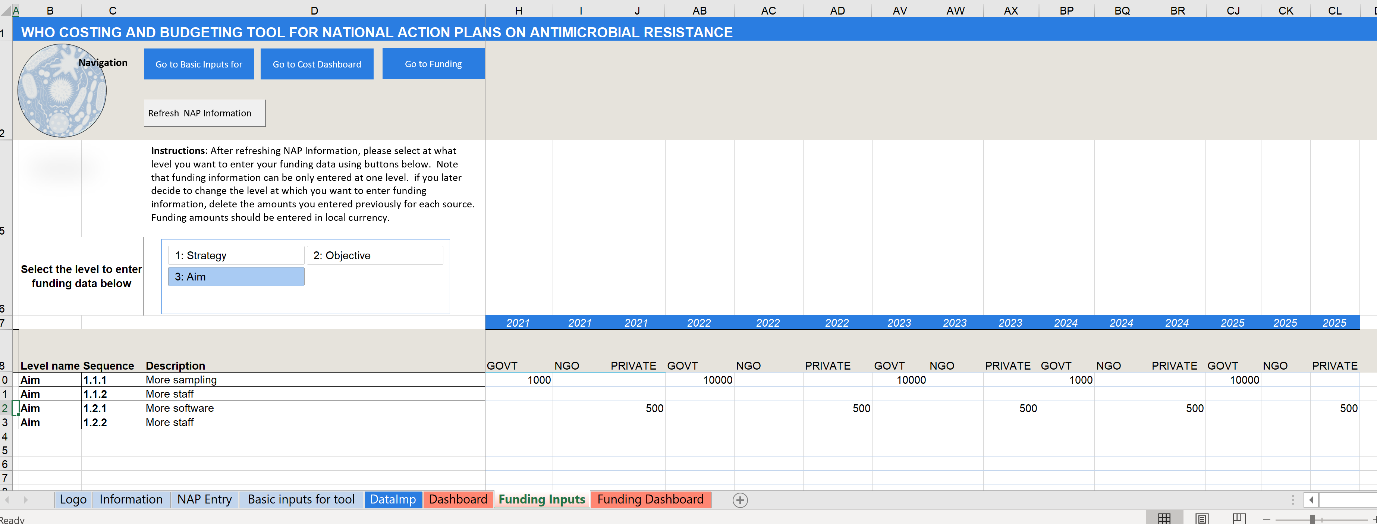


1. **Costing “Dashboard” output which costs by sector and NAP strategy’s objectives and aims**

*MOA – ministry of agriculture, MOE – ministry of environmental issues, MOH – ministry of Health*


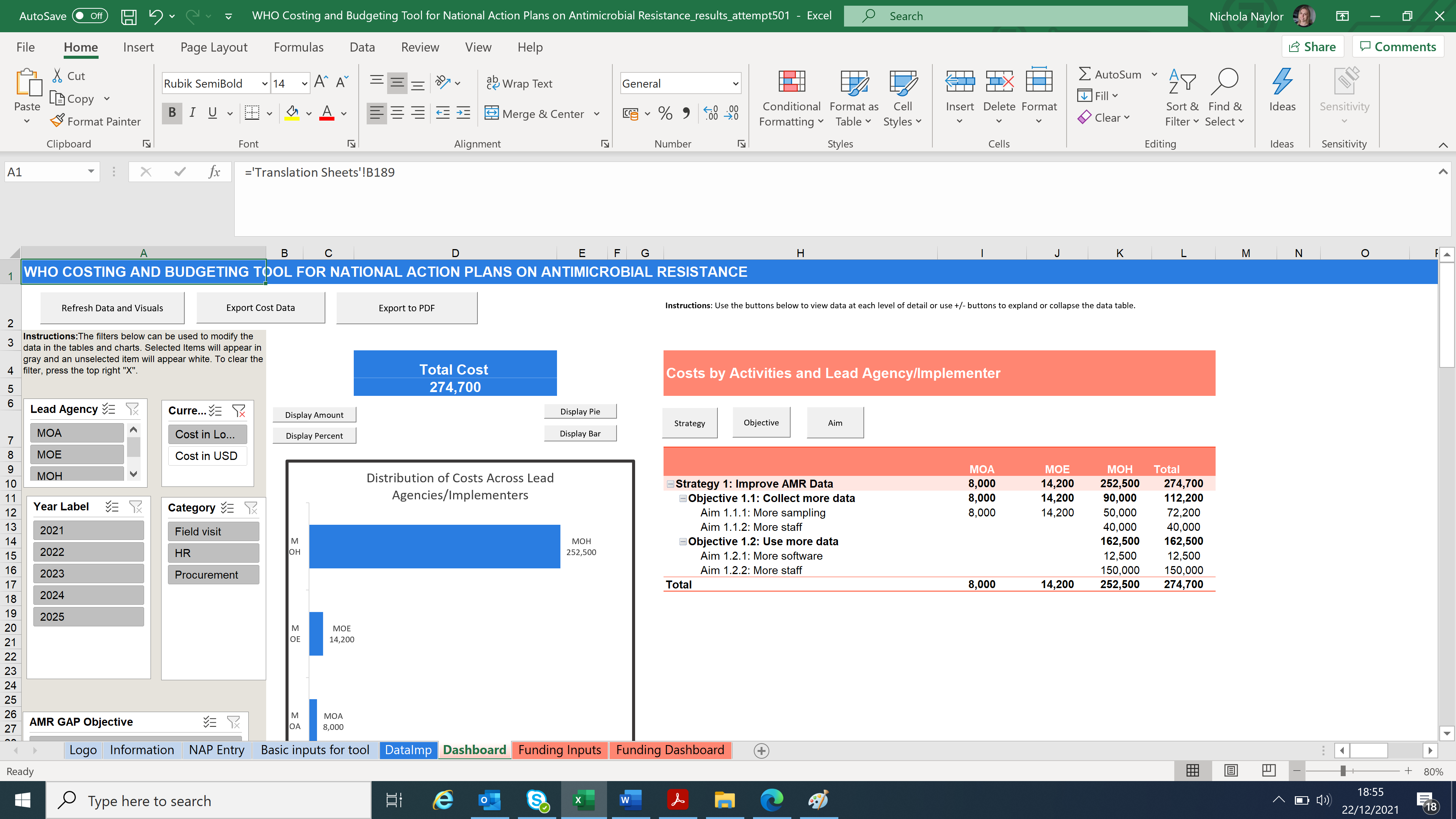


1. **Funding “Dashboard” Output which compares costs to allocated funding, flagging potential gaps.**


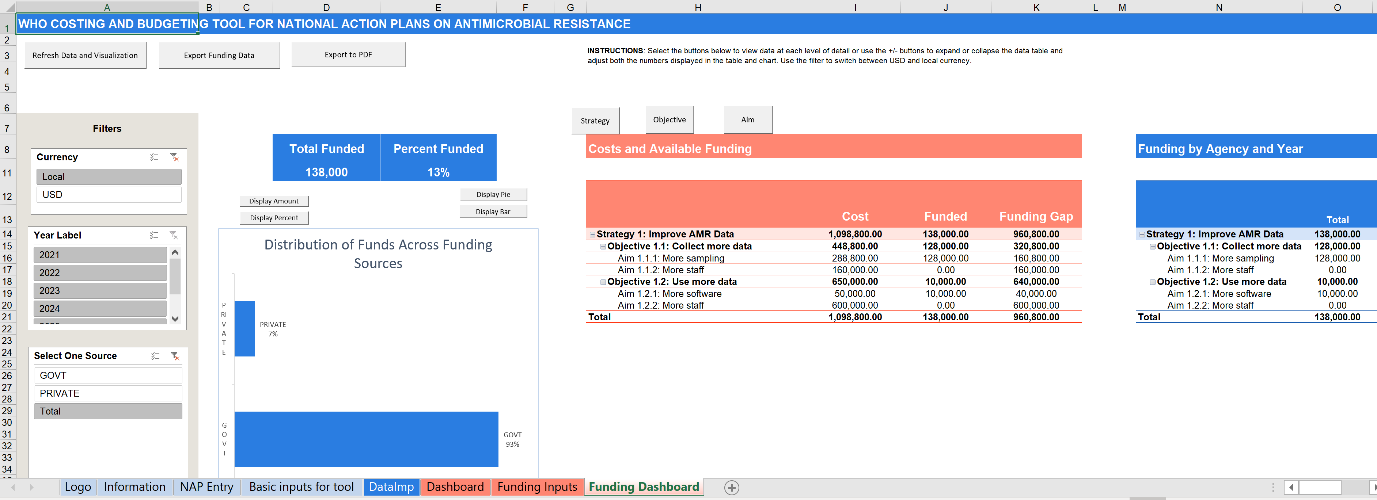


## Figure 2. The OneHealth Tool Interface


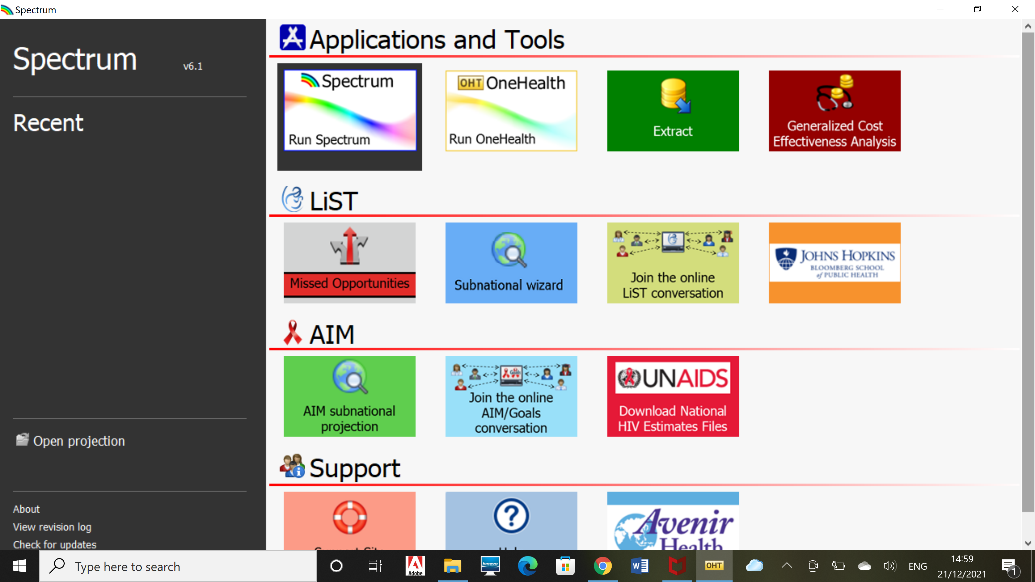


The OneHealth tool considers the whole health system costs and benefits of different policy interventions compared to different scenarios,(1) including the potential opportunity costs to other programmes (e.g. immunization vs others), to build cost-effective, efficient health service packages of interventions.(2) The tool illustrates the health system implications of scaling up intervention delivery, also allowing for a comparison of costs with the estimated financial resources available.

The tool is compatible with Microsoft Excel in that inputs and outputs can be copied across, however, this tool is its own downloadable software, hosted by Avenir. This means that the tool may be less flexible in terms of structure, and less transparent in terms of access of back-end, in comparison to tools built in Microsoft Excel or open-source software such as R.

This tool uses epidemiological modules (from Spectrum and its associated modules)(3) and health economic data (from the Generalized Cost-Effectiveness module) to create cost-effectiveness for a range of diseases and populations. In terms of infectious diseases, modules are available for; Malaria, HIV/Aids, Tuberculosis, certain sexually transmitted diseases and certain immunizations. Interestingly, the results of “TIME” (the TB focused module) are used to generate cost-effectiveness estimates of interventions using OneHealth, reporting in incident TB, including the number of multiple-drug resistant cases and the number of deaths due to TB, and cost and disability-adjusted life year impact of different intervention scenarios. We can see from the TrACss survey that many countries have AMR NAPs linked to existing action plans/strategies/targets in relation to Tuberclusis, Immunizations and other potential already existent modules.(4)

1. Avenir Health. OneHealth Manual: A system to create short and medium plans for health services [Internet]. [cited 2022 Oct 5]. Available from: https://avenirhealth.org/Download/Spectrum/Manuals/OneHealthManualE.pdf

2. Eregata GT, Hailu A, Stenberg K, Johansson KA, Norheim OF, Bertram MY. Generalised cost-effectiveness analysis of 159 health interventions for the Ethiopian essential health service package. Cost Eff Resour Alloc. 2021 Jan 6;19(1):2.

3. Avenir Health. Spectrum Manual: Spectrum System of Policy Models [Internet]. [cited 2022 Oct 5]. Available from: https://avenirhealth.org/Download/Spectrum/Manuals/SpectrumManualE.pdf

4. Global Database for the Tripartite Antimicrobial Resistance (AMR) Country Self-assessment Survey (TrACSS) [Internet]. 2022. Available from: https://amrcountryprogress.org/#/map-view
